# Selective Resection of Two Ipsilateral Supernumerary Hemivertebrae in a Child With Congenital Scoliosis: A Case Report

**DOI:** 10.7759/cureus.117518

**Published:** 2026-10-06

**Authors:** Bishnu Dev Sharma, Masato Tanaka, Tadashi Komatsubara, Ryo Ugawa, Kiran Gurung

**Affiliations:** 1 Orthopaedic Surgery, Okayama Rosai Hospital, Okayama, JPN; 2 Orthopaedic Surgery, Okayama University Hospital, Okayama, JPN

**Keywords:** congenital deformity, congenital spine disease, posterior spinal fusion and instrumentation, scoliosis surgery, surgical navigation

## Abstract

Management of congenital scoliosis with multiple hemivertebrae remains challenging. Clinical decisions should take into consideration various factors, such as the location, morphology, and growth potential of each hemivertebra, as well as overall spinal balance. Early surgery is typically recommended to halt the progression of deformity. Strategies include resection of the hemivertebra using a posterior or combined anterior-posterior approach, followed by fusion.

This report describes a case of a six-year-old child with three hemivertebrae on the same side, leading to an unbalanced spine that was progressive in nature. Preoperative planning showed three right-sided supernumerary hemivertebrae at the T6, T12, and L2 levels (supernumerary cervical, thoracic, and lumbar segments). The proximal thoracic curve due to the T6 hemivertebra was not progressive, but the T12 and L2 hemivertebrae produced thoracolumbar/lumbar curves and typically progressed over time. We planned selective surgical resection of the T12 and L2 vertebrae via a posterior approach and achieved a 93% correction of the major thoracolumbar/lumbar Cobb angle (57° to 4°). There was no neurological deterioration, and the child fared well after surgery. We believe this case will help us with clinical decision-making and surgical planning for similar cases.

## Introduction

Congenital scoliosis is a group of spinal deformities resulting from a vertebral anomaly that occurs during intrauterine spinal development. There can be failure of formation, failure of segmentation, or both. Hemivertebra is the most common cause of congenital scoliosis resulting from failure of formation, with a reported incidence of 3-10 per 10,000 live births [1]. The incidence of multiple hemivertebrae (three or more) ranges from 2.5% to 7.5% [2]. A hemivertebra usually replaces a normal vertebral structure or can be supernumerary. The literature search shows only a few reports on supernumerary hemivertebrae [3].

Understanding hemivertebral morphology is important because it determines the type of deformity that develops, the likelihood of progression, and the challenges of complete excision when surgery is planned. A hemivertebra can be fully segmented, semi-segmented, or non-segmented, depending on the presence of normal disk space on either side. When the anterior and posterior components of a hemivertebra correspond, it is said to be in unison; otherwise, it is discordant [4]. Multiple hemivertebrae, when present on the same side of the spinal column, will produce an unbalanced curve; continuous hemivertebrae on the same side, with one or more hemivertebra(e) on the contralateral side, will result in a partially balanced curve, whereas all hemivertebrae located on both sides, with no continuous hemivertebrae located on the same side, will completely balance the spinal column.

The rate of progression in unbalanced curves due to multiple vertebrae ranges from 2 to 5 degrees per year, with some cases reported as high as 29.3 degrees per year [5]. Such cases require surgery as early as possible to prevent the development of severe deformities. Treatment of hemivertebra is usually surgical, though some cases can be managed conservatively; bracing has a minimal role in congenital scoliosis and may be tried in cases awaiting surgery or to prevent the formation of secondary curves. The literature describes several surgical techniques: in situ fusion, convex hemi-epiphysiodesis, hemivertebra excision, and growing rods. Some authors favor a hybrid technique involving hemivertebrectomy with short fusion, along with traditional dual growing rods [6]. A current literature review of 22 studies on hemivertebra resection in young children advocates surgery before malformation becomes a deformity [7]. The first description of hemivertebra resection used a combined anterior and posterior approach for circumferential exposure, which was later modified to a single posterior approach [8].

We present a case of three ipsilateral supernumerary hemivertebrae with additional segmentation abnormalities and the decision to selectively resect only the two lesions considered responsible for progression.

## Case presentation

A six-year-old male child presented to us with complaints of progressive deformity of his back. His parents noticed a bump on his back at two months of age. He was initially managed at another hospital with a brace from the age of 1.5 years, but the deformity still progressed. He had a normal birth history, with no other siblings having a similar deformity. His height and weight were 112 cm and 17.6 kg, respectively. On examination, there was a right-sided rib hump with an elevated right shoulder, no skin pigmentation, no intellectual disability, and intact neurology.

Radiological evaluation showed right-sided thoracolumbar/lumbar scoliosis, with curve progression from 45 degrees (five months old) to 53 degrees (two years old), with some correction achieved with bracing (49 degrees at five years old) (Figure 1). On admission to our hospital (six years old), he was evaluated with whole-spine standing radiographs with side-bending and traction films (Figure 2). The Cobb's angle of the thoracolumbar/lumbar curve was 57 degrees, the proximal thoracic curve was 21 degrees, and there was a kyphosis of 2 degrees. The curve improved to 46 degrees on side bending and 37 degrees on traction. The pelvis showed no visible ossification of the iliac crest apophysis.

**Figure 1:**
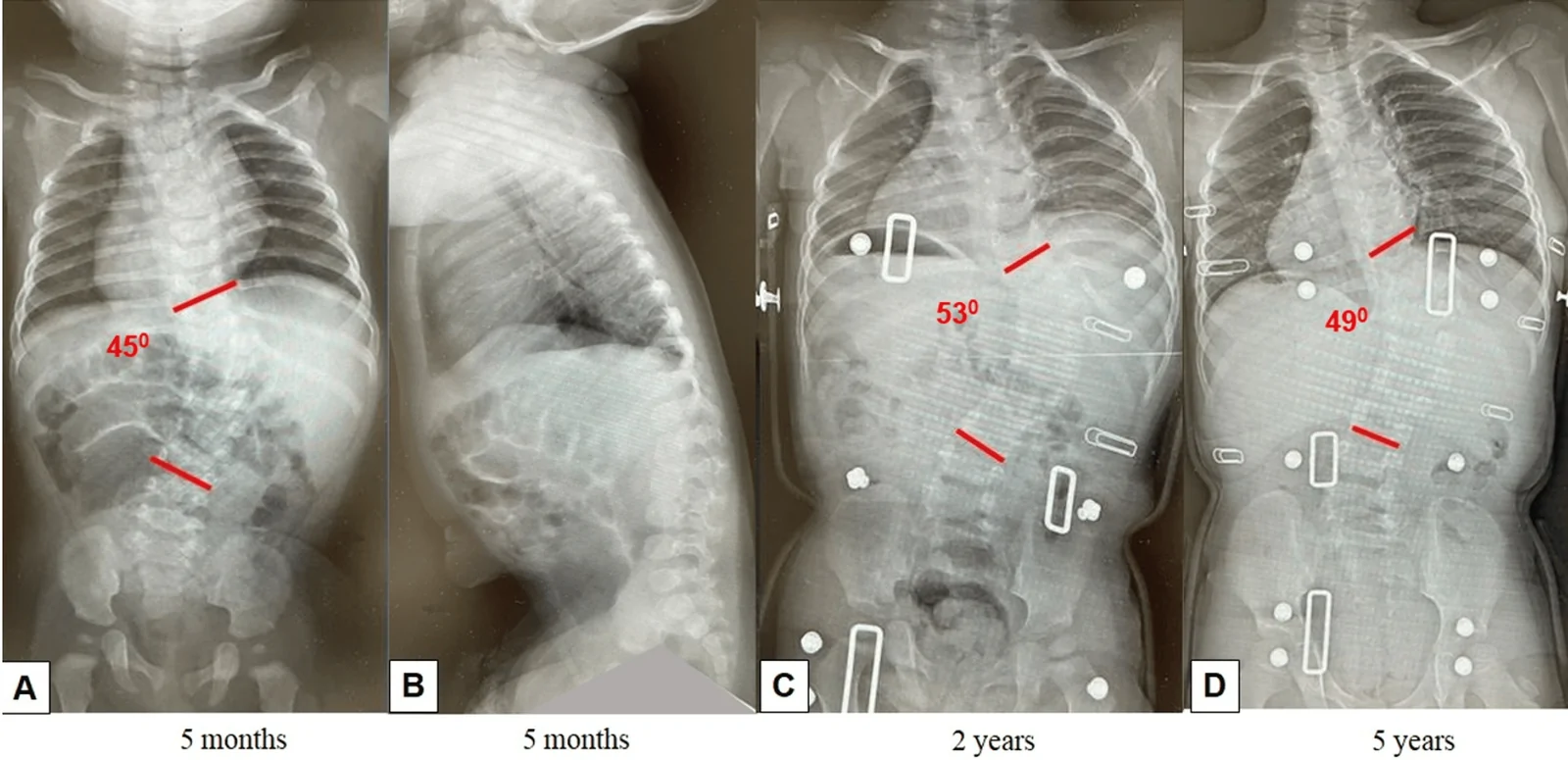
Chronological change of deformity A) Whole-spine posteroanterior radiogram at five months of age; B) Whole-spine lateral radiogram at five months of age; C) Whole-spine posteroanterior radiogram at two years of age; D) Whole-spine posteroanterior radiogram at five years of age with brace.

**Figure 2:**
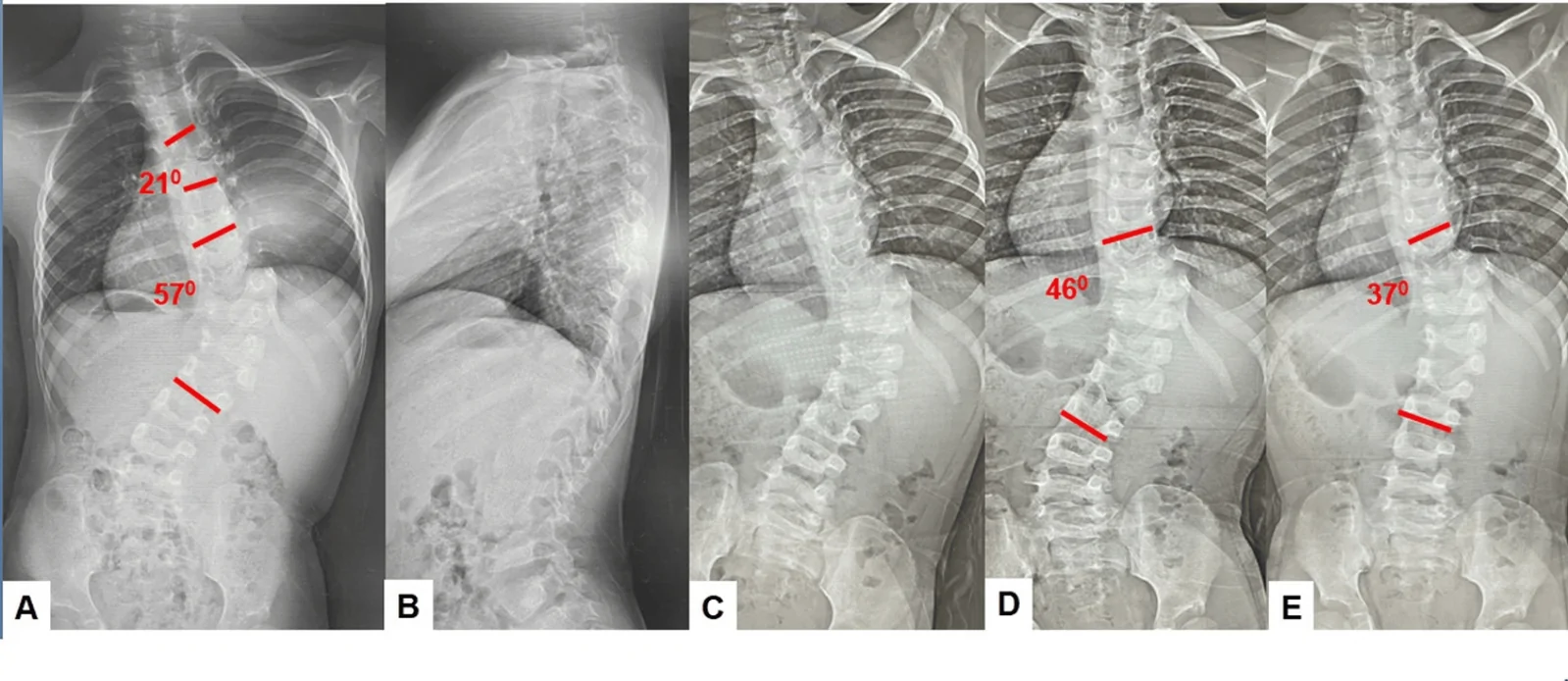
Preoperative radiological evaluation A) Whole-spine standing posteroanterior radiogram; B) Whole-spine standing lateral radiogram; C) Left-side-bending radiogram; D) Right-side-bending radiogram; E) Traction radiogram. The curve was corrected to 46 degrees on the left-side-bending radiogram and 37 degrees on the traction radiogram.

Computed tomography (CT) revealed the presence of three hemivertebrae at the T6, T12, and L2 levels (Figure 3). There were 8 cervical, 13 thoracic, and 6 lumbar vertebrae (multiple supernumerary vertebrae). All three hemivertebrae were present on the right side, leading to an unbalanced spine. The T6 hemivertebra was incarcerated and semi-segmented (fused superiorly with T5); T12 was non-incarcerated, fully segmented, and discordant (mismatch between the vertebral body and posterior components); and L2 was non-incarcerated and semi-segmented (fused superiorly with L1). There were also four butterfly vertebrae at the C4, C7, T2, and L1 levels and 13 ribs on the right side, with the first and second ribs fused, and 11 ribs on the left side. Magnetic resonance imaging (MRI) revealed no abnormality in the spinal cord. Urogenital, cardiac, and pulmonary evaluation showed no associated anomalies.

**Figure 3:**
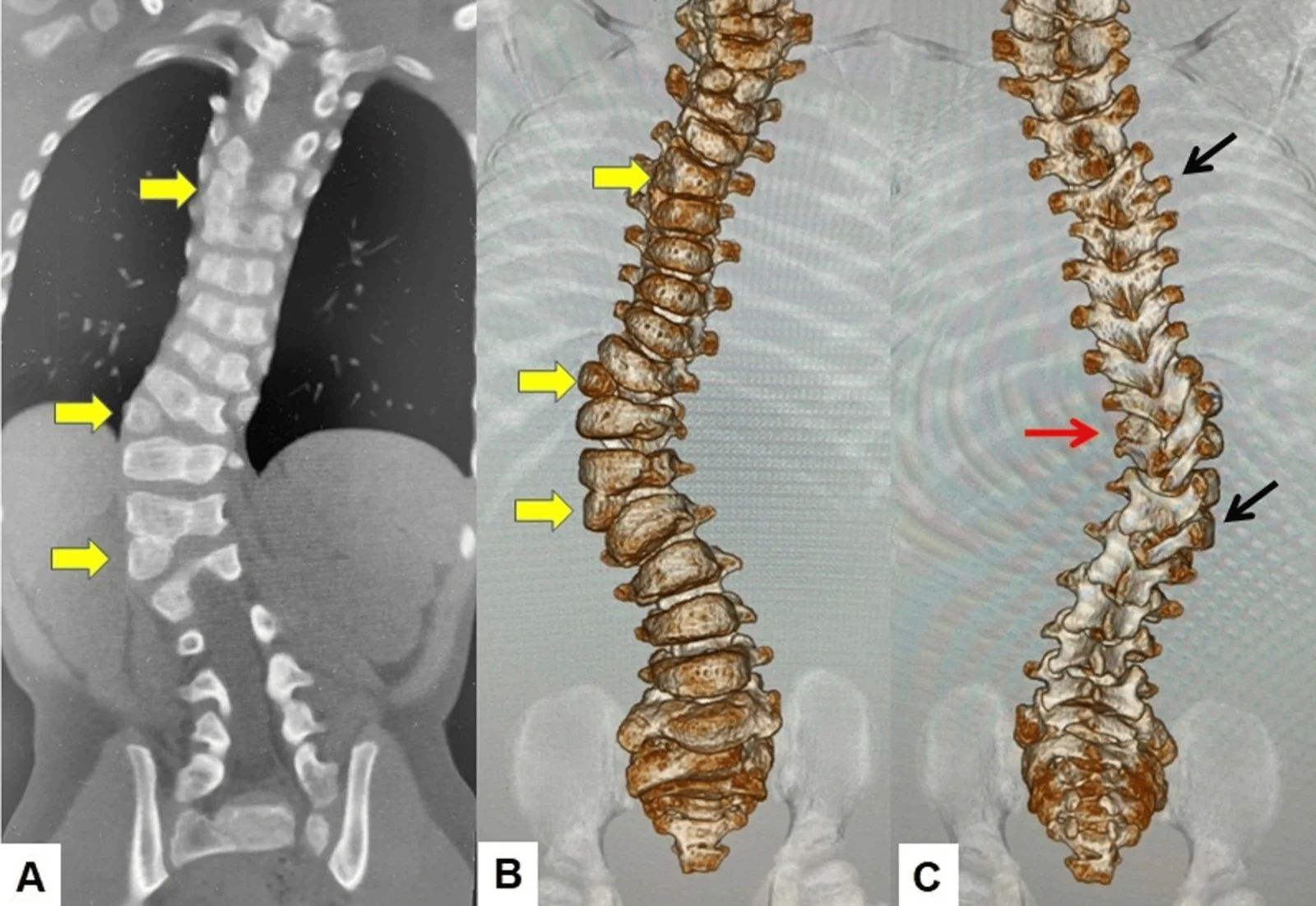
Preoperative CT A) Coronal reconstruction CT; B) anterior 3D CT; C) posterior 3D CT. 3D reconstruction CTs show right-sided multiple hemivertebrae at the T6, T12, and L2 levels (yellow arrows), T6 and L2 unison hemivertebrae (black arrows), and a T12 discordant hemivertebra (red arrow). CT: Computed Tomography

The child had a progressive deformity (57 degrees) with two non-incarcerated hemivertebrae in the thoracolumbar/lumbar region, one fully segmented and the other semi-segmented, and was planned for surgical resection and fusion. Not every hemivertebra necessarily requires resection in a child with multiple hemivertebrae. Therefore, the T6 hemivertebra, which was semi-segmented, incarcerated, and non-discordant, with a Cobb angle of 21 degrees, was less likely to progress and was thus not planned for surgical excision (Table 1).

**Table 1:** Radiological characteristics of the hemivertebrae

Level	Segmentation	Incarceration	Morphology	Contribution to deformity	Surgical decision
T6	Semi-segmented	Incarcerated	Non-discordant	Relatively limited proximal thoracic curve (21°)	Preserved
T12	Fully segmented	Non-incarcerated	Discordant	Major contributor to thoracolumbar deformity	Resected
L2	Semi-segmented	Non-incarcerated	Non-discordant	Major contributor to thoracolumbar/lumbar deformity	Resected

The patient was positioned prone with neuromonitoring attached. A midline skin incision was made from T11 to L3, and bilateral pedicle screws were inserted at the T11, T13, and L3 levels under O-arm navigation (reference frame placed on the L3 spinous process). Bone holes were created in both hemivertebrae, and marking pins were inserted. After removing the T11-L3 spinous processes, the intervertebral spaces were curetted, and the posterior vertebral lamina was removed using the Kerrison technique. Under navigation, the hemivertebrae were excavated using a high-speed drill (Midas; Medtronic, Minneapolis, MN, USA), followed by peeling soft tissues from the thinned outer walls of the vertebral bodies. Meticulous hemostasis was performed throughout the procedure. First, a connecting rod was attached on the left side and distracted, and then the right-sided rod was inserted and compressed to correct the deformity. Fusion was performed using resected autogenous bone. The operative time was 161 minutes, with an estimated blood loss of 460 mL; no blood transfusion was necessary.

Postoperatively, there was no neurological deterioration. Postoperative radiographs showed correction of the deformity to 4 degrees, with 93% correction of the Cobb's angle (Figure 4). The CT scan showed complete resection of both hemivertebrae and correct placement of the implants (Figure 5).

**Figure 4:**
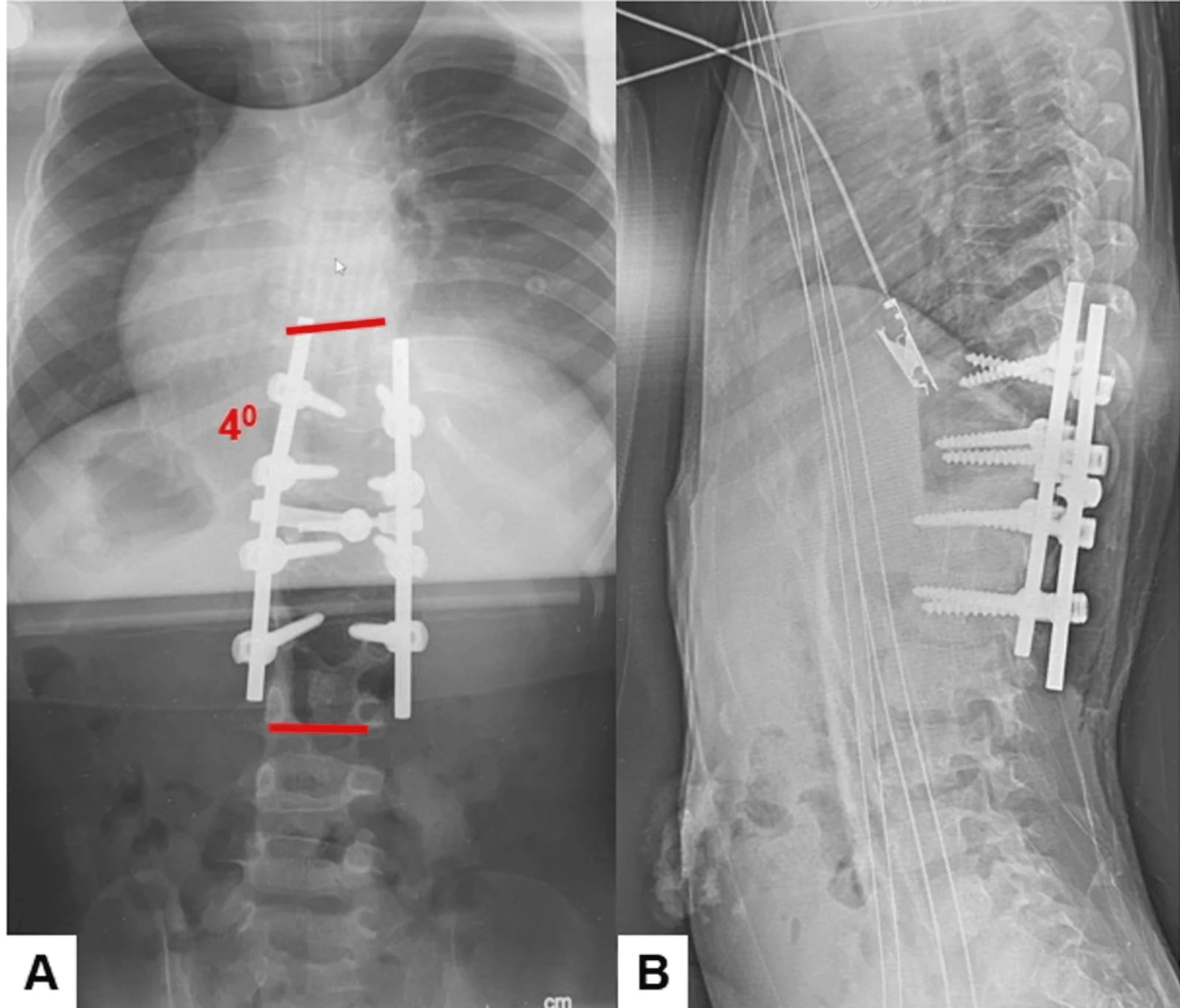
Postoperative radiograms A) Anteroposterior radiogram; B) Lateral radiogram. Anteroposterior radiogram shows correction of the deformity to 4 degrees.

**Figure 5:**
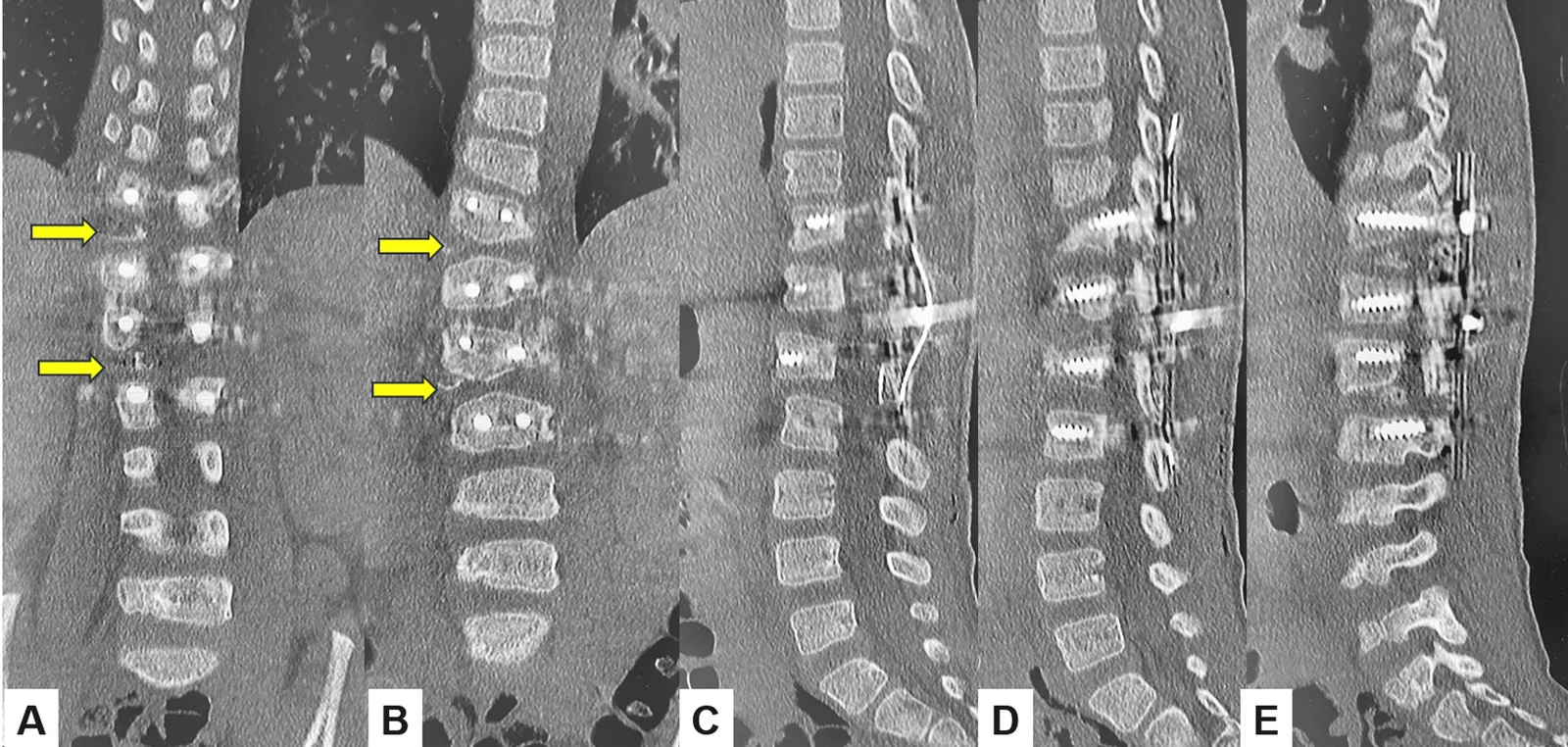
Postoperative CT A, B) Coronal reconstruction CTs; C-E) Sagittal reconstruction CTs. The two hemivertebrae were completely removed (yellow arrows). CT: Computed Tomography

His postoperative period was uneventful; he walked five days after surgery and was discharged two weeks after mobilization. He wore the soft brace for four months after surgery. At one-year follow-up, he is doing well with no neurological complications (Figure 6).

**Figure 6:**
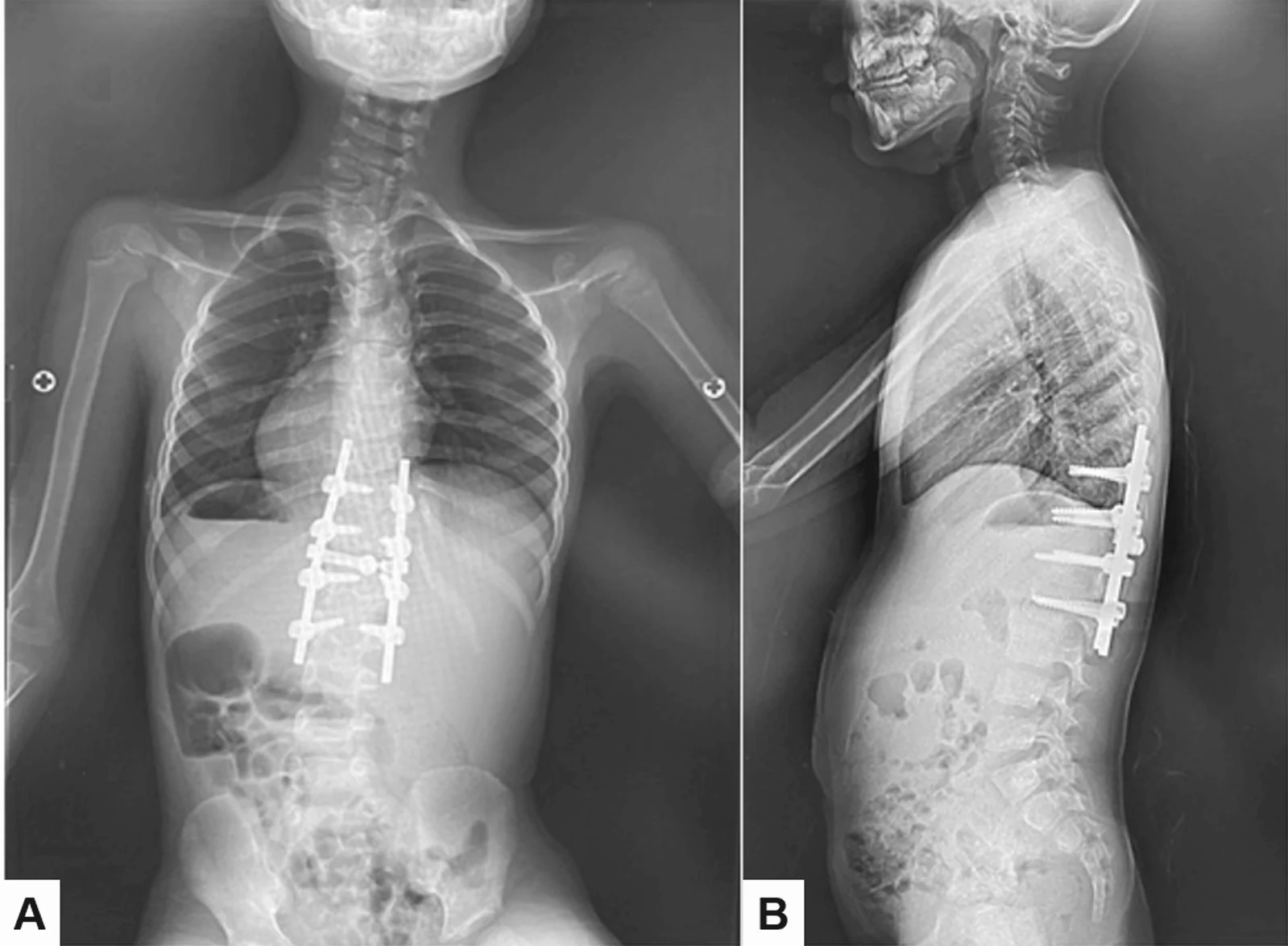
Follow-up radiograms at one year A) Whole spine standing posteroanterior radiogram; B) Whole spine standing lateral radiogram. Good spinal alignment was obtained without bracing.

## Discussion

The risk of deterioration of congenital scoliosis due to hemivertebrae depends upon the site, degree of segmentation, number of hemivertebrae, and spinal balance when multiple hemivertebrae are present. Thoracolumbar curves progress most rapidly, whereas upper thoracic curves progress more slowly but can cause shoulder imbalance and cosmetic deformity [2]. In patients with multiple hemivertebrae, the rate of curve progression is highest in unbalanced curves (12.1 degrees/year) compared with partially unbalanced (4.2 degrees/year) or completely balanced (3.6 degrees/year) curves [9]. All these factors should be considered when treating patients with multiple hemivertebrae. In our case, there were two hemivertebrae located in the thoracolumbar/lumbar region - one fully segmented and one semi-segmented - indicating a high risk of curve progression. Also, all three hemivertebrae were on the same side, producing an unbalanced curve. Studies have shown that it is not always necessary to remove all hemivertebrae; those likely to cause progressive deformity can be selectively removed [10]. This will help reduce surgical morbidity, surgical time, blood loss, and perioperative complications. We also performed selective removal of two hemivertebrae at the thoracolumbar/lumbar location that caused the major structural deformity, whereas the thoracic hemivertebra, which had very little influence on the curve and the overall coronal balance, was left unresected.

Congenital scoliosis with a single hemivertebra is straightforwardly managed with resection and short fusion. Children with multiple hemivertebrae need detailed planning with an individualized approach. Various approaches to hemivertebra excision have been described in the literature, with the current trend toward a single posterior approach [11]. Combined anterior-posterior approaches have higher morbidity and complication rates, up to 42% as reported in the literature [12]. Hemivertebra resection via a posterior approach has lower morbidity and no difference in correction rate compared with the combined anterior and posterior approach [13].

Various authors have reported correction rates after hemivertebra excision using a posterior-only approach, ranging from 48% to 84% [13,14]. We achieved a 93% correction in our case, with 460 mL of blood loss and 161 minutes of surgical time. In the study by Guo et al., the blood loss was 306.6 mL, and the operative time was 186.4 minutes [14]. Mladenov et al., in their retrospective comparative study between single posterior and combined anterior and posterior approaches, found similar curve correction (59% vs. 55%), but a decreased duration of surgery (272 vs. 319 minutes) and no surgery-related general complications (0 vs. 38%) in the single posterior approach [15]. In another systematic review and meta-analysis including 37 studies, the mean operative time was 227 minutes and mean blood loss was 522 mL for the posterior-only approach, which were significantly lower than those for the combined approach (operative time, 316 minutes; blood loss, 888 mL); however, no difference was noted in the curve correction rate (66% vs. 69%) [16]. We performed a posterior-only approach and limited short fusion in our case.

The age at which to excise a hemivertebra remains debated. Early surgery may achieve better correction with fewer fusion segments but is challenging due to low bone density, small pedicles, and anesthesia-related concerns. A recent review article found no agreement on the ideal age and suggests a threshold of 18 months [7], while others suggest delaying surgery until three to five years of age [17]. Our child was initially managed at another center and presented to us only at the age of six years, when surgery was performed.

With the introduction of computer-assisted navigation to improve pedicle screw placement accuracy, the use of navigation in complex spine surgeries is expanding [18]. A few case reports have utilized this technique for hemivertebra resection [19]. In our case, O-arm navigation may have resulted in shorter operative time and reduced blood loss compared with previously reported studies [16]. Complex morphological variations, such as a discordant hemivertebra, may complicate complete excision. Intraoperative neuromonitoring provides continuous, multimodal assessment of the spinal cord and nerve roots to minimize postoperative neurological deficits [20]. We had no neurological deterioration postoperatively.

Limitations

The main limitation of this report is its single-case design, which limits the generalizability of the surgical strategy. In addition, longer-term follow-up is required to assess the maintenance of correction, spinal growth, fusion status, and the potential development of junctional deformity or compensatory curves.

## Conclusions

Selective hemivertebra resection may be considered for patients with multiple hemivertebrae, depending on the location, type, overall spinal balance, and likelihood of progression. A posterior-only approach with limited fusion, guided by navigation, achieved effective short-term correction without neurological complications in this patient, while emphasizing the need for longer follow-up.
